# Identification of Ossnrk1a−1 Regulated Genes Associated with Rice Immunity and Seed Set

**DOI:** 10.3390/plants13050596

**Published:** 2024-02-22

**Authors:** Yingying Cao, Minfeng Lu, Jinhui Chen, Wenyan Li, Mo Wang, Fengping Chen

**Affiliations:** 1 Fujian Universities Key Laboratory for Plant-Microbe Interaction, Ministerial and Provincial Joint Innovation Centre for Safety Production of Cross-Strait Crops, College of Life Science, Fujian Agriculture and Forestry University, Fuzhou 350002, China; caoyy0906@163.com (Y.C.); luminfeng2021@163.com (M.L.); 1210514007@fafu.edu.cn (J.C.); 1220514102@fafu.edu.cn (W.L.); 2 State Key Laboratory for Conservation and Utilization of Bio-Resources in Yunnan, Yunnan Agricultural University, Kunming 650201, China; 3 Key Laboratory of Biopesticides and Chemical Biology, Ministry of Education, Fujian Agriculture and Forestry University, Fuzhou 350002, China

**Keywords:** rice, OsSnRK1a, seed set, resistance, blast fungus, gene expression

## Abstract

Sucrose non-fermenting–1-related protein kinase–1 (SnRK1) is a highly conserved serine–threonine kinase complex regulating plants’ energy metabolisms and resistance to various types of stresses. However, the downstream genes regulated by SnRK1 in these plant physiological processes still need to be explored. In this study, we found that the knockout of *OsSnRK1a* resulted in no obvious defects in rice growth but notably decreased the seed setting rate. The *ossnrk1a* mutants were more sensitive to blast fungus (*Magnaporthe oryzae*) infection and showed compromised immune responses. Transcriptome analyses revealed that SnRK1a was an important intermediate in the energy metabolism and response to biotic stress. Further investigation confirmed that the transcription levels of *OsNADH-GOGAT2*, which positively controls rice yield, and the defense-related gene *pathogenesis-related protein 1b* (*OsPR1b*) were remarkably decreased in the *ossnrk1a* mutant. Moreover, we found that OsSnRK1a directly interacted with the regulatory subunits OsSnRK1β1 and OsSnRK1β3, which responded specifically to blast fungus infection and starvation stresses, respectively. Taken together, our findings provide an insight into the mechanism of OsSnRK1a, which forms a complex with specific β subunits, contributing to rice seed set and resistance by regulating the transcription of related genes.

## 1. Introduction

Throughout their life cycle, plants are constantly confronted with various biotic and abiotic stresses. The former consists of pathogenic microorganisms (fungi, bacteria, and nematodes), insects, and herbivores, as well as nutrient deficiency, extreme temperature, salinity, drought, floods, etc. To survive, plants have evolved sophisticated coping mechanisms to perceive stressful stimuli and activate the necessary cellular responses, including the activation of various kinase cascades [1], transcriptional reprogramming [2], reactive oxygen species (ROS) production [3], and the accumulation of defense-related hormones [4].

Sucrose non-fermenting–1 (SNF1)-related protein kinases (SnRKs), initially isolated from rye endosperm [5], are conserved Ser/Thr kinase complexes that have been manipulated to enhance the performance of eukaryotes on starvation stress [6]. The SnRK1 complex contains a catalytic α subunit and regulatory β and γ subunits, with each SnRK1 subunit having multiple isoforms [7,8]. The SnRK families, diverging in different kingdoms, evolved into three subfamilies (SnRK1, SnRK2, and SnRK3) in plants [9]. The SnRK1 family is highly conserved among species and is homologous to SNF1 in fungi and AMP-activated protein kinase (AMPK) in mammals [10]. However, the SnRK2 and SnRK3 families are considered specific to plants and participate in stress signaling pathways [11,12,13].

Plant SnRK1s are known to be involved in the regulation of growth, metabolism, hormone signaling, stress responses, and gene expression [14,15,16]. SnRK1 regulates sucrose availability by activating transcription factors that induce amylase expression and by regulating key enzymes in sucrose synthesis [16,17]. It also promotes catabolism and inhibits anabolism through transcriptional and post-translational regulation in response to starvation via glucose sensing and signaling [16,18,19,20,21]. Phosphorylation modification, mediated by SnRK1, has a direct impact on several processes related to the transport of non-structural carbohydrates, including starch degradation, sucrose metabolism, phloem transport, sugar transport across flattened bodies, and glycolysis [22,23,24,25]. Besides the energy metabolism, mounting evidence shows that plant SnRK1s play important roles in plants’ defense against various pathogens [26,27]. For example, SnRK1a has been reported to enhance barley’s resistance against the fungal pathogen *Blumeria graminis* f. sp. *hordei* (*BgH*) (which causes powdery mildew) [28], limit geminivirus infection in crops [29], and improve defense against *Plasmodiophora brassicae* (which causes clubroot disease) in *Arabidopsis thaliana* [30].

Rice (*Oryza sativa* L.) is one of the most vital global staple crops, and its global cultivated area reached 165 million ha. in 2021 (https://www.fao.org/faostat/en/#data/QCL; last accessed on 17 February 2024). Nevertheless, rice yield is affected by many factors, among which diseases such as rice blast, rice blight bacteria, and false smut disease caused by *Magnaporthe oryzae* (*M. oryzae*), *Xanthomonas oryzae* pv. *oryzae* (*Xoo*), and *Ustilaginoidea virens*, respectively, are the main biotic factors decreasing the yield and quality [31]. The rice genome encodes three SnRK1a kinases, which can be classified into two subgroups: SnRK1a, containing OsSnRK1a (OSK1), and SnRK1b, containing OSK24 and OSK35 [32]. Through gene overexpression and silencing, *OsSnRK1a* was found to positively regulate the growth and broad-spectrum resistance of rice [33], and OSK35 was also found to be a positive regulator in rice’s defense against *M. oryzae* and *Xoo* [34]. In addition, OsSnRK1a maintains its conserved function in rice sugar signaling [16] and source–sink communication [15]. However, the downstream genes in the different rice signaling pathways regulated by OsSnRK1a are still unknown. Therefore, to explore the regulatory crosstalk of OsSnRK1a in rice immune resistance and energy metabolism, we created *OsSnRK1a*–knockout mutants using CRISPR/Cas9-mediated gene editing and performed transcriptome analyses to identify the OsSnRK1a-regulated genes involved in the crop’s defense and seed set. Moreover, the potential roles of different regulatory β subunits associated with OsSnRK1a in response to stress were investigated.

## 2. Results

### 2.1. Deletion of OsSnRK1a Decreased Rice Seed Setting Rate

**Figure 1:**
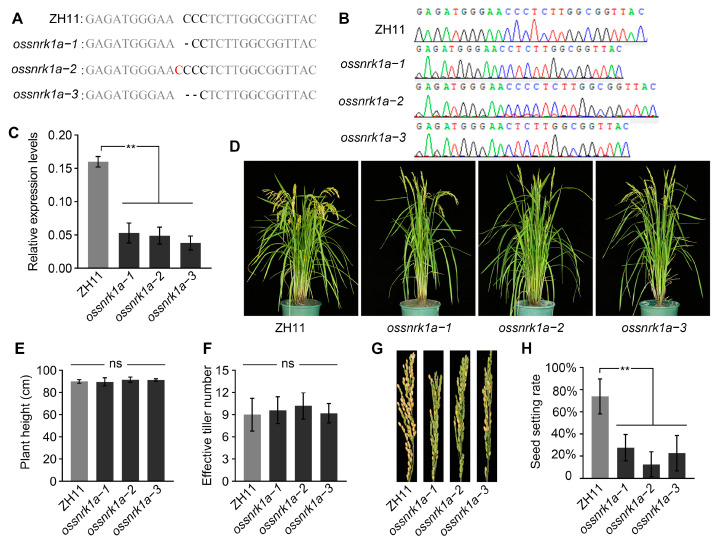
Knockout of *OsSnRK1a* led to a decrease in rice seed setting rate. (**A**,**B**) Mutation sites of the *ossnrk1a* allelic mutants created by CRISPR/Cas9-mediated gene editing. (**C**) The expression of *OsSnRK1a* mRNA in three mutants compared to ZH11. (**D**) Field-grown plants of ZH11 and *ossnrk1a* mutants were photographed at the mature stage. (**E**,**F**) The *ossnrk1a* mutants displayed similar plant heights and numbers of effective tillers to the wild type. Bars represent mean ± standard deviation (SD) (*n* = 5). According to Student’s *t*-test, “ns” indicates non-significance. (**G**) Mature panicles of ZH11 and *ossnrk1a* mutants. (**H**) Seed setting rates of ZH11 and the *ossnrk1a* mutants, with bars representing mean values ± SD (*n* = 5). Statistically significant differences indicated by ** (*p* < 0.01, Student’s *t*-test).

To better determine OsSnRK1a’s biological functions in rice, we knocked out *OsSnRK1a* using CRISPR-associated nuclease 9 (Cas9)-mediated gene editing in the rice japonica cultivar Zhonghua11 (ZH11) and obtained three mutants from the independent T0 transgenic lines (Figure 1A,B). The *ossnrk1a*−*1* and *ossnrk1a*−*2* mutations carried a 1 bp deletion and insertion, respectively, while *ossnrk1a*−*3* harbored a 2 bp deletion in the *OsSnRK1a* coding region. All the three allelic mutations resulted in a frame shift in the *OsSnRK1a* ORF. We further confirmed that the expression of *OsSnRK1a* in all three ossnrk1a mutants was significantly lower than in ZH11 (Figure 1C).

When grown in the field until the mature period, the *ossnrk1a* mutants (*ossnrk1a*−*1*, *ossnrk1a*−*2*, and *ossnrk1a*−*3*) showed no obvious defects in plant height or effective tiller number at maturity (Figure 1D–F). However, at the maturity stage, all three *ossnrk1a* mutants displayed significantly decreased seed setting rates (Figure 1G,H) and spikelet numbers per spike (Figure S1E) compared to the wild type. Meanwhile, we found that there were no significant differences in the grain weight and size between *ossnrk1a* mutants and ZH11 (Figure S1). These results indicate that OsSnRK1a plays an important role in rice seed set and spikelet numbers per spike under normal growth conditions. In addition, when subjected to continuous darkness and starvation for 4 days, we found that *ossnrk1a*−*1* displayed greater stress (with the leaves demonstrating more severe chlorosis) compared with the wild type (Figure S2), which is consistent with the conserved role of plant SnRK1a in response to sugar starvation and survival in extended darkness [14,35].

### 2.2. Knockout of OsSnRK1a Undermined Rice Resistance to Blast Fungus

**Figure 2:**
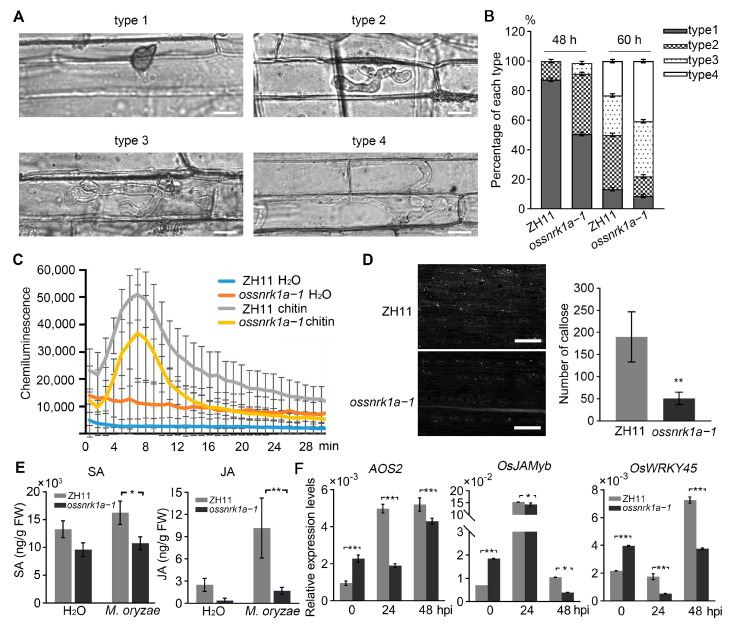
OsSnRK1a positively regulates rice resistance against blast fungus. (**A**) Inoculation of ZH11 and *ossnrk1a*−*1* leaf sheaths with Guy11 conidia. Four invasive hyphae (IH) types were observed during the infection process (type 1, with no hyphal penetration; type 2, with primary IH formation; type 3, with secondary IH formation; type 4, with IH extending to the neighboring cells). Scale bar = 50 μm. (**B**) Percentage of the four IH types in ZH11 and *ossnrk1a*−*1* cells was measured at 48 hpi and 60 hpi. Error bars represent SD (*n* = 3). (**C**) Detection of ROS burst generated from ZH11, and *ossnrk1a*−*1* leaf discs were treated with 400 nm chitin at the indicated time points. Error bars represent the SD (*n* = 8, technical replicates). This assay was performed in three independent replicates with similar results (Supplemental File S2). (**D**) Observation of callose deposition on ZH11 and *ossnrk1a*−*1* leaves after chitin treatment (left), bars = 100 µm. The number of callose depositions (bright white dots) was counted using ImageJ (right, Bethesda, MD, USA), bars represent mean values ± SD from five independent microscopy fields. Statistically significant differences were indicated by Student’s *t*-test (** *p* < 0.01). These data are from one of three independent experiments with similar results (Supplemental File S2). (**E**) Quantification of endogenous JA and SA levels. Data are means ± SD from five biological replicates, with each containing five individual plants. (**F**) Relative transcript levels of the defense-related genes in ZH11 and *ossnrk1a*−*1* before and after spraying inoculation with Guy11 conidia. Data are means ± standard error (SD) (*n* = 3). Significant differences were determined by Mann–Whitney test (* *p* < 0.05; ** *p* < 0.01). The qRT-PCR assays were performed in three independent replicates with similar results (Supplemental File S2).

Previous studies have reported that OsSnRK1a contributes to broad-spectrum disease resistance in rice [33]. We inoculated three-week-old *ossnrk1a* allelic mutant seedlings (*ossnrk1a*−*1*, *ossnrk1a*−*2*, and *ossnrk1a*−*3*) with *M. oryzae* isolate Guy11 conidial spores via spraying and found that the *ossnrk1a* mutants developed more numerous and larger diseased lesions on their leaves compared to the ZH11 seedlings (Figure S3A). Measurement of the lesion area revealed that the knockout of OsSnRK1a increased the susceptibility of the rice to the blast fungus (Figure S3B). To quantify this susceptibility in the absence of OsSnRK1a, we carried out punch inoculation assays (Figure S3C). The relative fungal biomass (*MoPot2*/*OsUBQ*) within the infected regions showed that the *ossnrk1a* mutants supported significantly more blast fungus growth compared to ZH11 (Figure S3D), although there seemed to be reduced fungal length in some mutants. Furthermore, a rice leaf sheath inoculation assay was carried out to monitor the *M. oryzae* invasion process in *ossnrk1a*−*1* and ZH11. The statistical results at 48 and 60 h post inoculation (hpi) indicated that invasive hyphae (IH) of the blast fungus indeed extended faster in *ossnrk1a*−*1* leaf sheath cells (Figure 2A,B). Taken together, our data confirm that OsSnRK1a acts as a positive regulator in rice’s defense against *M. oryzae*.

To better understand the defective immune responses in the absence of OsSnRK1a upon blast fungus infection, we first measured the ROS burst from 7-day-old *ossnrk1a*−*1* and ZH11 seedlings upon chitin treatment. At the tested time points, the *ossnrk1a* mutant produced lower ROS burst levels than the ZH11 (Figure 2C). In addition, we investigated callose deposition on ZH11 and *ossnrk1a*−*1* leaves after chitin treatment and found that *ossnrk1a*−*1* generated significantly fewer callose plugs (bright white dots) than ZH11 (Figure 2D). These results suggest that the knockout of *OsSnRK1a* compromises rice’s PAMP-triggered immunity (PTI) responses and confirm that OsSnRK1a play an important role in immune responses upon fungus infection. Moreover, the contents of the defensive hormones salicylic acid (SA) and jasmonic acid (JA) were measured in ZH11 and *ossnrk1a*−*1*. It was found that at 48 hpi with *M. oryzae* conidial spores via spraying, *ossnrk1a*−*1* displayed significantly lower levels of SA and JA than the wild type (Figure 2E). The transcriptional levels of the defense-related genes were investigated. *Allene oxide synthase 2* (*AOS2*) encodes a key enzyme in the JA biosynthetic pathway, which converts 13-hydroperoxylinolenic acid to 12,13-epoxylinolenic acid [36]. OsWRKY45 is essential for SA signaling-induced resistance against rice pathogens such as *M. oryzae* and *Xanthomonas oryzae* pv. *oryzae* (*Xoo*) [37,38]. OsJAMyb has been reported to play roles in JA-mediated abiotic and biotic stress responses in rice [39]. Consistent with the decreased levels of SA and JA in the absence of OsSnRK1a, the transcription of all the three genes was significantly lower in *ossnrk1a*−*1* than those in ZH11 following blast fungus inoculation (at 24 and 48 hpi) (Figure 2F). Taken together, our data indicate that OsSnRK1a contributes to blast fungus resistance by positively regulating rice PTI responses and SA/JA-mediated defensive signaling pathways.

### 2.3. Transcriptome Analyses Showing Defective OsPR1b Induction by M. oryzae Infection in ossnrk1a−1

**Figure 3:**
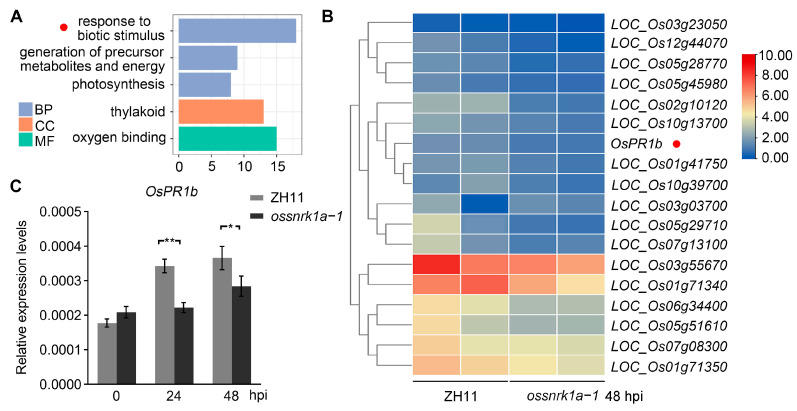
Transcriptome and qRT-PCR analyses revealed that the knockout of *OsSnRK1a* caused a defect in *OsPR1b* induction by *M. oryzae* infection. (**A**) GO term enrichment analyses of the down-regulated genes in *ossnrk1a*−*1* compared to ZH11 at 48 hpi. The red dot indicates the term where putative defense-related genes are enriched. BP, biological process; MF, molecular function; CC, cellular component. (**B**) Heat map of genes enriched in the term of response to biotic stimulus. *OsPR1b* is indicated by a red dot. (**C**) Relative transcription levels of *OsPR1b* in ZH11 and *ossnrk1a*−*1* before and after *M. oryzae* infection. Bars present means ± SD (*n* = 3). Significant differences were determined via Mann–Whitney test (* *p* < 0.05; ** *p* < 0.01). These data are from one of three independent experiments with similar results (Supplementary File S2).

To further investigate the genome-wide transcriptional changes resulting from OsSnRK1a deletion, we first performed transcriptome deep sequencing (RNA-seq) of *ossnrk1a*−*1* and ZH11 leaves harvested without Guy11 inoculation (mock treatment) and 48 hpi Guy11 inoculation. As shown in Figure S4A, the two biological replicates for RNA-seq displayed a clear correlation. The differently expressed genes (DEGs) were defined as upregulated or downregulated genes with a |fold-change| > 2 between *ossnrk1a*−*1* and ZH11. There were 454 and 73 genes consistently upregulated or downregulated, respectively, in *ossnrk1a*−*1* compared to ZH11, regardless of mock treatment or *M. oryzae* inoculation (Figure S4B,C). To determine which defense-related genes had their induction by *M. oryzae* infection positively regulated by OsSnRK1a, we conducted a Gene Ontology (GO) enrichment analysis with the downregulated genes at 48 hpi in *ossnrk1a*−*1* and compared the results with ZH11, allowing us to identify terms related to various biological processes (BPs), cellular components (CCs), and molecular functions (MFs). One biological process, namely, to respond to biotic stimuli (marked by the red dots in Figure 3A), was enriched with eighteen genes (Table S2), including *pathogenesis-related protein 1b* (*OsPR1b*) (Figure 3B, red dots). OsPR1b is a rice defense-related gene specifically triggered by JA/SA or blast fungus infection [40,41]. We further confirmed the results using a qRT-PCR assay and found that without *M. oryzae* invasion, the transcription level of *OsPR1b* was higher in *ossnrk1a*−*1* than in the wild type, whereas at 24 and 48 hpi with Guy11, *OsPR1b* was upregulated in ZH11 but not in the *ossnrk1a* mutant, resulting in a significantly lower *OsPR1b* transcription in *ossnrk1a*−*1* compared to that in ZH11 (Figure 3C). Thus, OsSnRK1a contributes to the induction of defense-related gene *OsPR1b* following *M. oryzae* invasion.

### 2.4. OsSnRK1a Is a Positive Regulator in OsNADH-GOGAT2 Expression

**Figure 4:**
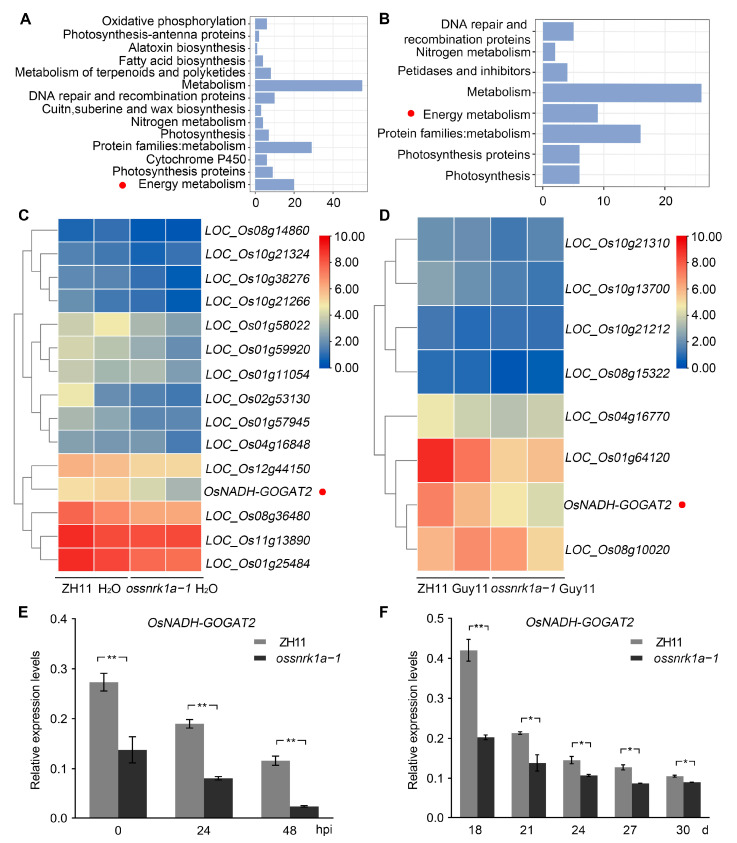
KEGG enrichment and qRT-PCR analyses indicate that *OsNADH-GOGAT2* is downregulated in the *ossnrk1a* mutant. (**A**,**B**) KEGG enrichment analyses of the downregulated genes in *ossnrk1a*−*1* compared to ZH11 before and after inoculation with *M. oryzae*. Red dots indicate the energy metabolism pathways, where *OsNADH-GOGAT2* was enriched. (**C**,**D**) Heat maps of the genes enriched in the term of energy metabolism from (**A**,**B**), respectively. *OsNADH-GOGAT2* is indicated by red dots. (**E**) The qRT-PCR assays indicate relative transcript levels of *OsNADH-GOGAT2* in ZH11 and *ossnrk1a*−*1* before and after inoculation with *M. oryzae*. (**F**) Relative transcript levels of *OsNADH-GOGAT2* in *ossnrk1a*−*1* are significantly lower than those in ZH11 at different growth stages. Bars in (**E**,**F**) represent means ± SD (*n* = 3) with significant differences denoted as * *p* < 0.05 and ** *p* < 0.01 via Mann–Whitney test. These data are from one of three independent replicates with similar results (Supplementary File S2).

When analyzing the RNA-seq data of ZH11 and the *ossnrk1a* mutant, energy metabolism terms were enriched via KEGG analysis in the downregulated genes in *ossnrk1a*−*1* relative to ZH11 with or without *M. oryzae* infection (Figure 4A,B, red dot; Table S2). One consistent gene, *OsNADH-GOGAT2*, was identified (Figure 4C,D, red dot) in the energy metabolism terms. *OsNADH-GOGAT* was mainly found expressed in completely expanded leaves and leaf sheaths during the vegetative stage, and its knockout mutant resulted in a severe reduction in rice yield when grown in a paddy field [42]. The decreasing transcript levels of *OsNADH-GOGAT2* in the *ossnrk1a* mutant were confirmed via a qRT-PCR assay performed using *ossnrk1a*−*1* and ZH11 leaf tissues without and with *M. oryzae* inoculation (Figure 4E). Moreover, we analyzed the transcription of *OsNADH-GOGAT2* in the leaves of *ossnrk1a*−*1* and ZH11 at different growth stages and found that the *OsNADH-GOGAT2* transcripts were constantly kept at a significantly lower level in the *ossnrk1a* mutant compared with the wild type (Figure 4F). The results suggest that the expression of *OsNADH-GOGAT2* was compromised in the absence of OsSnRK1a, which is consistent with the reduction in the seed setting rate observed in the *ossnrk1a* mutants.

### 2.5. OsSnRK1a Interacts with the Regulatory Subunits OsSnRK1β1 and OsSnRK1β3

**Figure 5:**
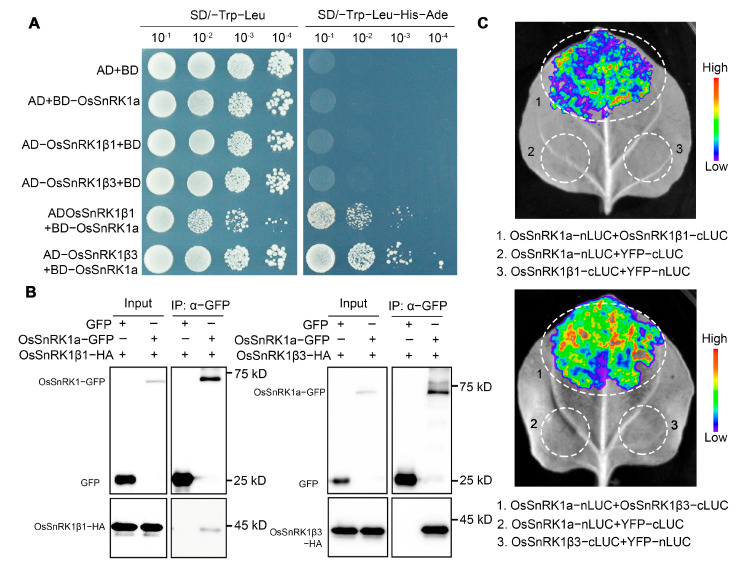
OsSnRK1a interacts with the β subunits, including OsSnRK1β1 and OsSnRK1β3. (**A**) Direct interaction between OsSnRK1a and OsSnRK1β1/3, determined via Y2H assay. The combinations involving the empty vectors AD or BD were utilized as negative controls. (**B**) In vivo association between OsSnRK1a and OsSnRK1β1/3 was determined using Co-IP assays via Agrobacterium-mediated transient expression in *Nicotiana benthamiana* (*N. benthamiana*) leaves. The combinations with GFP co-expression were employed as the negative controls. The IP was carried out with anti-GFP beads, and Western blotting was performed with anti-GFP or anti-HA tag antibodies. (**C**) Split-luciferase complementation assays showed that OsSnRK1a can form complexes with OsSnRK1β1 or OsSnRK1β3. The OsSnRK1β subunits and OsSnRK1a fused with the C-terminal (cLUC) halves and OsSnRK1a fused with the N-terminal (nLUC) halves of luciferase, respectively, were transiently co-expressed in *N. benthamiana* leaves. The combinations with nLUC or cLUC were used as the negative controls.

To further identify the regulatory β subunits forming a complex with OsSnRK1a, we successfully cloned the full length cDNA of two β subunits in ZH11, named *OsSnRK1β1* (LOC_Os03g20340) and *OsSnRK1β3* (LOC_Os05g41220). To determine the interaction between SnRK1a and OsSnRK1β1/3, we first carried out yeast two-hybrid (Y2H) assays, which indicated that both OsSnRK1β subunits directly interacted with OsSnRK1a (Figure 5A). Furthermore, the Co–IP (with GFP transient expression as negative control) and split-LUC complementation (with transient expression of YFP fused with nLUC or cLUC as negative control) assays were performed, which showed that SnRK1a associates with OsSnRK1β1 and OsSnRK1β3 in vivo (Figure 5B,C). Based on these findings, we concluded that SnRK1a can form complexes with either OsSnRK1β1 or OsSnRK1β3.

### 2.6. Transcriptional Pattern Analysis for the SnRK1 Subunits

**Figure 6:**
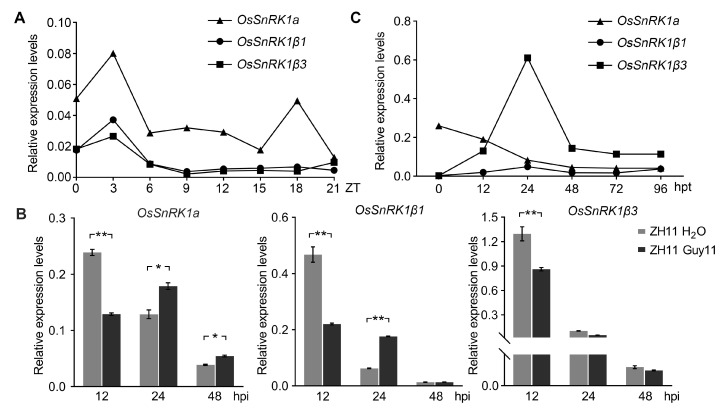
The transcriptional patterns of *OsSnRK1a*, *OsSnRK1β1*, and *OsSnRK1β3* in response to blast fungus invasion and starvation stresses. (**A**) Expression patterns of *OsSnRK1a*, *OsSnRK1β1*, and *OsSnRK1β3* in 3-week-old ZH11 at different time points of a diel cycle. (**B**) Transcript levels of the genes in ZH11 with or without inoculation with *M. oryzae*. For the mock treatment, 0.02% Tween−20 water was used. Bars represent means ± SD (*n* = 3). Significant differences were determined by Mann–Whitney test (* *p* < 0.05, ** *p* < 0.01). (**C**) Expression patterns of *OsSnRK1a*, *OsSnRK1β1*, and *OsSnRK1β3* in response to starvation and darkness stress. The ZH11 leaf tissues were harvested for qRT-PCR analyses at the indicated time points during the continuous darkness treatment. All the data in this figure were from one of three independent replicates with similar results (Supplementary File S2).

To determine the spatial expression patterns of *OsSnRK1a*, *OsSnRK1β1*, and *OsSnRK1β3*, we harvested leaf, young panicle, and root tissues from ZH11 plants at the heading stage for qRT-PCR analysis. As shown in Figure S5, *OsSnRK1a*, *OsSnRK1β1*, and *OsSnRK1β3* displayed similar relatively high and low transcription levels in leaf and root tissues, respectively. To investigate their circadian expression patterns, we analyzed the relative transcriptional levels of *OsSnRK1a*, *OsSnRK1β1*, and *OsSnRK1β3* in the leaves of 3-week-old ZH11 seedlings harvested every three hours over a complete day and night cycle. The transcription levels of all three genes were raised at 6 h after lights off (Figure 6A, 3:00 a.m.). In addition, *OsSnRK1a*, but not *OsSnRK1β1* or *OsSnRK1β3*, was upregulated at 9 h after lights on ZT (Zeitgeber time) (Figure 6A, 6:00 p.m.).

In addition, we found that at the early stage of infection (12 hpi), the transcript levels of *OsSnRK1a*, *OsSnRK1β1*, and *OsSnRK1β3* were repressed by *M. oryzae* (Figure 6C). However, beginning at 24 hpi, *OsSnRK1a* transcription was upregulated by the blast fungus invasion, and similarly, *OsSnRK1β1* was induced at 24 hpi, whereas *OsSnRK1β3* transcription was not alerted by *M. oryzae* infection after 12 hpi (Figure 6C). Interestingly, *OsSnRK1β3*, rather than *OsSnRK1a* or *OsSnRK1β1*, was dramatically induced by the continuous darkness treatment (Figure 6C). Taken together, our data suggest that *OsSnRK1a* and *OsSnRK1β1* were induced by *M. oryzae* challenge, whereas *OsSnRK1β3* was specifically upregulated by darkness and starvation stress.

## 3. Discussion

SnRK1, a key protein kinase complex composed of multiple subunits, has functions in diverse plant physiological processes. Besides being known as a conserved energy sensor, SnRK1 also contributes to plant resistance against various pathogens [27,43]. However, the mechanism of plant SnRK1′s engagement in the regulation of different physiological processes in response to exogenous stresses is still unknown. In this study, via phenotypic analysis of the *OsSnRK1*–knockout mutants, we concluded that OsSnRK1a plays a positive role in rice seed set under normal growth conditions and immune responses upon rice blast fungus invasion.

The knockout of *OsSnRK1a* did not affect rice growth under normal growth conditions but did cause a reduction in seed setting rate. In addition, we observed that the *ossnrk1a*−*1* leaves displayed more severe chlorosis compared to the wild type under extended darkness, indicating that OsSnRK1a deletion enhanced rice’s sensitivity to starvation stress. Through our transcriptome analysis, we discovered that SnRK1a plays an important role as an intermediate in the sugar signaling cascade reaction (energy metabolism and photosynthesis) and nitrogen metabolism. Interestingly, transcription levels of the nitrogen metabolism enzyme encoding gene, *NADH-GOGAT*, is downregulated in the absence of OsSnRK1a, and it was constantly kept at a significantly lower level in the *ossnrk1a* mutant under normal growth conditions, indicating that OsSnRK1a is a positive regulator of *OsNADH-GOGAT2* transcription. It has been reported that disruption of *OsNADH-GOGAT2* gene expression resulted in a significant decrease in spikelet number per spike, as well as a reduction in yield and overall plant biomass [42,44]. Our study showed similar results (Figure S1E). Thus, our data suggest that *OsNADH-GOGAT2* is a downstream target regulated by OsSnRK1a in controlling rice seed set.

OsSnRK1a contributes to resistance against rice blast fungus. This resistance is associated with OsSnRK1a’s positive regulation of PTI responses and the transcription of defense-related genes. The knockout of OsSnRK1a compromises chitin-induced ROS burst and callose deposition in rice, as well as the induction of *AOS2*, *OsWRKY45*, and *OsJAMyb* upon *M. oryzae* infection. Furthermore, the transcriptome analysis revealed that OsSnRK1a is a positive regulator in the upregulation of *OsPR1b* induced by *M. oryzae* invasion. It was previously found that overexpression of *OsSnRK1a* resulted in raised JA levels and positively affected the SA pathway after *M. oryzae* inoculation [33]. Plant metabolic (sugar) status can affect hormone signaling [7,45]. Considering SnRK1′s function as a key regulator of the primary metabolism, its positive role in regulating rice immune responses may be partially implemented through the metabolic processes it controls. Meanwhile, we found that the defense-related genes were induced in the *ossnrk1a* mutant during the resting state (without *M. oryzae* infection). This may be because the absence of OsSnRK1 disrupts rice’s energy metabolism during normal growth, resulting in an endogenously stressed status, which causes the induction of defense-related genes. Together, these results suggest the complexity of OsSnRK1′s participation in rice’s innate immunity.

SnRK1 regulates gene expression through the phosphorylation of key enzymes to control metabolic processes. For example, in sucrose synthesis, sucrose phosphate synthase (SPS), a crucial enzyme in this pathway, is directly inactivated by the phosphorylation of SnRK1 [46]. During sugar starvation, OsSnRK1a can activate *MYBS1*, which, as a transcription factor, can upregulate amylase expression in rice embryos [16]. *OsMYBS1* is involved in regulating sugar and α-amylase gene expression in rice [47]. Moreover, the overexpression of SnRK1a in Arabidopsis cells triggers an increased expression of numerous ATG8 proteins. These proteins are essential for the development of autophagosomes and serve as highly sensitive indicators for both autophagy and carbon status [48]. Additionally, SnRK1 acts as a negative regulator of MYB75. The protein encoded by MYB75 redirects the carbon flow away from anthocyanin production towards the earlier stages of phenylpropanoid synthesis and other flavonoids. This shift in carbon allocation has the potential to impact cell wall composition, particularly the presence of lignin, and the transport of auxins [49,50]. Therefore, the increasing number of identified SnRK1-targeting transcription factors sheds light on how SnRK1 controls plant energy metabolism via transcriptional regulation of the relative genes. However, these transcription factors, as OsSnRK1 substrates controlling the expression of rice defense-related genes, need future studies to be identified.

The distal C-terminal subdomains of SnRK1a interact with the β and γ subunits. It is widely acknowledged that the functional SnRK1 kinase exists as a complex comprising a catalytic subunit associated with regulatory β and γ subunits. Additionally, free subunits may also play complementary roles in enhancing SnRK1 functions [51]. OsSnRK1a directly binds to the β subunits OsSnRK1β1 and OsSnRK1β3. *OsSnRK1β3* transcription was dramatically increased under continuous darkness, whereas the transcription levels of *OsSnRK1a* decreased gradually and *OsSnRK1β1* transcription in rice stayed almost unchanged under continuous darkness, implying that OsSnRK1β3 is mainly engaged in responding to starvation stress. In addition, upon blast fungus infection, *β1* rather than *β3* was dramatically induced. These results suggest that OsSnRK1a may need variable β subunits to form different OsSnRK1 complexes to respond to various stresses. However, the exact mechanism of the β subunits regulating OsSnRK1a activity in various biological processes still requires further study.

## 4. Materials and Methods

### 4.1. Plant Materials and Growth Conditions

We used CRISPR/Cas9 gene-editing technology to knock out *OsSnRK1a* (LOC_Os05g45420) from ZH11 [52]. The selected gRNA target sequence of *OsSnRK1a* is 5′-gcagagatgggaaccctcttgg-3′. To measure agronomic traits, the plants were grown in field in Fuzhou during the summer season. To investigate the responses to infection and starvation, germinated rice seeds were cultivated in a controlled environment chamber (Conviron, Winnipeg, MB, Canada, PGC20) at 28 °C with a 12 h photoperiod (600–800 μmol/m^2^·s) and 70% relative humidity.

### 4.2. Gene Expression Analyses

The rice leaves were subjected to RNA extraction using TRIzol reagent (Ambion, Austin, TX, USA, Cat. No. 15596018) following the manufacturer’s protocol. Subsequently, cDNA synthesis was performed using the *Evo M*–*MLV* RT kit (Accurate Biology Co., Ltd., Changsha, China, Cat. No. AG11711). For qRT-PCR analysis, MonAmp^TM^ Chemo HS qPCR Mix (Monad Biotech Co., Ltd., Wuhan, China, MQ00401S) was utilized in conjunction with the CFX96 Touch Real-Time PCR Detection System (Bio-Rad Laboratories, Inc., Irvine, CA, USA). The relative expression levels were calculated using the 2^−∆∆Ct^ method, with *OsUBQ* (LOC_Os03g13170) serving as the reference gene.

### 4.3. Blast Fungus Inoculation

Rice was inoculated with the *M. oryzae* isolate Guy11 following a previously described procedure [53]. Briefly, Guy11 was cultured on CM medium for 7 days and then transferred onto a rice bran medium for sporulation. A conidial suspension with 2 × 10^5^ spores/mL was sprayed onto the 3-week-old rice seedlings. After leaving the inoculated plants in the dark at 26 °C with 90% humidity for the first 24 h, they were transferred into a growth chamber at 28 °C with a 12 h photoperiod (600–800 μmol/m^2^·s) and 70% relative humidity and were sprayed with tap water 2–3 times a day. The diseased leaves were imaged at 3~5 days post inoculation. Then, the percentage of the lesion area was calculated using ImageJ.

Punch inoculation and fungal biomass investigations were conducted using six- to eight-week-old rice plants. The leaves on the plants were punctured on the front side distance from the vein. Then, the wounded site was inoculated with a drop of 1 × 10^5^ *M. oryzae* spores. The drop was maintained in place using transparent tape. The inoculated plants were placed in the chamber with 90% humidity at 26 °C for 5 to 7 days, and then the inoculated leaves were cut and photographed, following the previously described protocol [54]. Specifically, the DNA from infected leaf tissues was extracted, and the relative level of the fungal gene *MoPot2* was determined using *OsUBQ* as a reference gene.

### 4.4. Measurement of SA and JA Contents

Measurement of the JA and SA levels was conducted using 3-week-old *ossnrk1a*−*1* and ZH11 seedlings inoculated with 5 × 10^5^ Guy11 conidial spores/mL (spraying with water containing 0.02% Tween 20 was used as the mock treatment). The leaf tissues from five plants were harvested as one biological replicate. The methods of SA/JA extraction and quantification used were the same as previously described in [54].

### 4.5. Rice Sheath Inoculation Assays

A 200 µL conidial suspension bearing 1 × 10^5^ spores/mL of *M. oryzae* was infiltrated into the leaf sheaths of three-week-old seedlings of the *ossnrk1a* mutant and ZH11. The seedlings were kept in a chamber at 28 °C and under high humidity. The progress of infection in sheath cells was observed under a microscope (40× objective, Zeiss LSM880, ZEISS VISION CENTER, Freiburg, Germany) at 48 and 60 hpi. Fifty conidial spores were counted as one replicate, and three replicates were analyzed for each type.

### 4.6. ROS Burst Detection

The method for measuring the chitin-induced ROS burst levels is described in [54]. Briefly, leaf discs were cut from 7-day-old rice seedlings and floated on water overnight, and then the water was removed. After that, the leaf discs were incubated with 100 µL reaction solution (20 µM luminal, Sigma Aldrich, Saint Louis, MO, USA, Cat. No. 123072 and 2.5 µg/mL peroxidase, Solarbio Science & Technology Co., Ltd. Beijing, China, Cat. No. P8020) containing 400 nM chitin (Sigma-Aldrich, Saint Louis, MO, USA, Cat. No. H1396) to immediately detect the ROS burst. The luminescence was measured by a Berthold Mithras luminometer (Berthold Technologies GmbH & Co.KG, Bad Wildbad, Germany) every 1~2 min for 55 min. Eight replicates were performed for each sample.

### 4.7. Callose Deposition Assay

For the observation and quantitative assessment of chitin-induced callose deposition, leaves of the 7-day-old seedlings on a 1/2 Murashige and Skoog (Beyotime Biotechnology, Co., Ltd., Haimen, China, Cat. No. B5008S) plate were detached and subjected to treatment with 400 nM of chitin. The tissues were fixed with a fixative solution (ethanol:glacial acetic acid = 3:1) for at least 5 h, and three fixative changes were ensured. Subsequently, they were immersed in 70% ethanol for 2 h, followed by immersion in 50% ethanol for an additional 2 h, and left overnight in water. After three washes with ddH_2_O, the tissues were treated with 10% NaOH for transparency purposes over a duration of one hour and then washed four times with ddH_2_O. Finally, the tissues were stained using a solution of 0.01% aniline blue (dissolved in pH 9.5 KH_2_PO_4_ at a concentration of 150 mM) for four hours. The more detailed method was described previously in [55]. The callose depositions in the leaves were photographed using confocal microscopy under fluorescence excitation with a wavelength of 340~380 nm (Zeiss LSM880, ZEISS VISION CENTER, Freiburg, Germany). The number of deposits was counted using ImageJ (Bethesda, MD, USA) [56]. The results from five independent microscopy fields were subjected to statistical analysis.

### 4.8. Yeast Two-Hybrid Analyses

The coding sequences of *OsSnRK1a*, *OsSnRK1β1*, and *OsSnRK1β3* were amplified and cloned into pGADT7 or pGBKT7 (Clontech), as indicated. Subsequently, the resulting constructs were co-transformed into yeast strain AH109. The obtained yeast clones grown on synthetic defined (SD) medium lacking Trp and Leu (SD–Trp–Leu) were then spotted onto SD medium lacking Trp, Leu, His, and Ade (SD–Trp–Leu–His–Ade) at 30 °C for 3 days to detect the interactions.

### 4.9. Split-Luciferase Complementation Assay

The coding regions of *OsSnRK1a*, *OsSnRK1β1*, and *OsSnRK1β3* were cloned to nLUC or cLUC vectors, as indicated in [54]. Then, the fusion proteins were transiently co-expressed in *N. benthamiana* leaves via agroinfiltration (*Agrobacterium tumefaciens*, strain GV3101). After three days, the infiltrated leaves were detached and treated with 1 mM precooled luciferin (Beyotime, China, Cat. No. ST198) and then kept in the dark for 5~10 min. The combinations with nLUC or cLUC were used as the negative controls. The LUC luminescence signals were imaged using the LB985 Night SHADE (Berthold, Germany) with a CCD imaging system.

### 4.10. Co-IP Assays

To determine the interaction between OsSnRK1a and OsSnRK1β1 or OsSnRK1β3 in plants using co-IP assays, OsSnRK1a–GFP was co-expressed with OsSnRK1β1-HA or OsSnRK1β3-HA via Agrobacterium-mediated transient expression in *N. benthamiana* leaves. The total proteins were extracted from the leaf tissues harvested at three days after agroinfiltration using an extraction buffer (50 mM Tris-HCl pH7.5, 150 mM NaCl, 1 mM EDTA, 1 mM DTT, 1% IGEPAL CA-630, 10% glycerol, 1mM PMSF, 1× protease inhibitor cocktail). Then, 10 μL GFP-Nanoab-Agarose beads (Lablead Trading Co., Ltd., Beijing, China, GNA−20–400) were added to each protein sample, incubated at 4 °C, and softly shaken for 2 h. Then, the beads were washed four times with a washing buffer (i.e., the extraction buffer with 0.3% IGEPAL CA630) and then resuspended with 80 μL of washing buffer. After adding 20 μL of 5 × SDS loading buffer, the samples were subjected to Western blotting with anti-GFP (TransGen Biotech Co., Ltd., Beijing, China, Cat. No. HT801) and anti-HA antibodies (Abmart Shanghai Co., Ltd., Shanghai, China, Cat. No. M20003S). Chemiluminescence was detected using Pierce ECL substrate (ThermoFisher Scientific lnc., Waltham, MA, USA) and the ChemiDoc^TM^ Imaging system (Bio-Rad Laboratories, Inc., Irvine, CA, USA) [57].

### 4.11. Transcriptome Deep Sequencing and Data Analysis

Three-week-old ZH11 and *ossnrk1a*−*1* seedlings were subjected to spraying inoculation with *M. oryzae* conidia (0.02% Tween-20 solution was sprayed as a mock control). Seven or eight leaves of ZH11 or *ossnrk1a*−*1* were collected as one of three biological replicates for each treatment. Total RNA extraction, mRNA-seq library construction, and sequencing were carried out as described previously [54] using the leaf tissue samples, which were then sent to Beijing Novo. The mRNA-seq library was prepared and sequenced as described previously, with minor modifications. The mRNA enriched in total RNA was treated with DNase I to completely remove genomic DNA contamination. The mRNA was purified using magnetic beads conjugated with oligo poly-T. The mRNA was further purified using the AMPure XP system (Beckman Coulter, Beverly, CA, USA) to synthesize and screen cDNAs 370–420 bp in size to construct the mRNA-seq library [58]. The final libraries were quality-controlled and sequenced on the Illumine platform. For RNA sequencing data analysis, the raw sequence data (reads) were trimmed using Trimmomatic (v0.39) to remove spliced and low-quality reads. The paired-end clean reads were compared to the reference genome using Hisat2. The number of reads mapped to each gene was calculated using featuarrets (v1.6.4). The FPKM for each gene was then calculated based on the length of the gene, and the number of reads mapped to that gene was calculated. Gene expression differences between the two comparative combinations were detected using DESeq2 R software (v1.30.1) (each group contained two biological replicates). The genes exhibiting *p* < 0.05 and absolute log_2_ (fold-change) > 2 were designated as differentially expressed. The GO enrichment analysis toolkit and database (AgriGo v2.0, http://systemsbiology.cau.edu.cn/agriGOv2/index.php; last accessed on 17 February 2024) were used to analyze the biological processes, cellular components, and molecular functions of genes.

### 4.12. Starvation Treatment of Seedlings

Three-week-old ZH11 and *ossnrk1a*−*1* seedlings grown in the nutrient solution were transferred to water culturing under continuously dark conditions for 4 days. During this period, the tap water used for culturing was changed daily, and the samples were harvested at the indicated time points.

### 4.13. Statistical Analyses

The significant differences in plant height, seed setting rate, numbers of effective tillers, and callose deposition were determined using Student’s *t*-tests. Statistically significant differences are indicated by * (*p* < 0.05) and ** (*p* < 0.01). Significant differences in relative transcript levels of the examined genes were determined using a Mann–Whitney test (* *p* < 0.05; ** *p* < 0.01).

### 4.14. Primers and Plasmid Constructs

All of the primers and constructs used in this study are listed in Table S1.

### 4.15. Gene Accession Numbers

The sequences of the related genes in this study can be accessed in the Rice Genome Annotation Project database (http://rice.uga.edu/; last accessed on 17 February 2024) using the following accession numbers: *OsSnRK1a* (LOC_Os05g45420), *OsSnRK1β1* (LOC_Os03g20340), *OsSnRK1β3* (LOC_Os05g41220), *AOS2* (LOC_Os03g12500), *OsWRKY45* (LOC_Os05g25770), *OsJAMyb* (LOC_Os11g45740), *OsPR1b* (LOC_Os01g28450), *OsNADH-GOGAT2* (LOC_Os05g48200), and *OsUBQ* (LOC_Os03g13170).

## 5. Conclusions

By characterizing a series of *OsSnRK1a*–knockout mutants, we found that OsSnRK1a contributes to rice seed set and immune responses against blast fungus. Transcriptomic analysis revealed the role SnRK1a plays as an important regulator in rice energy metabolism and in the crop’s response to biotic stress. The consistent downregulation of *OsNADH-GOGAT2* likely leads to the decreased seed setting rates of the *ossnrk1a* mutants. OsSnRK1a deletion compromises chitin-induced ROS burst, callose deposition, and induction of defense-related genes such as *AOS2*, *OsWRKY45*, *OsJAMyb*, and *OsPR1b*, resulting in increased susceptibility to *M. oryzae* infection. Moreover, SnRK1a directly interacts with the regulatory β subunits OsSnRK1β1 and OsSnRK1β3. Finally, blast fungus infection and starvation stress were shown to upregulate *OsSnRK1β1* and *OsSnRK1β3* transcription, respectively. Taken together, our data show that OsSnRK1a contributes to defense against various stresses through upregulating the relevant genes in related pathways and by associating with the specific regulatory subunits.

## Data Availability

The original contributions presented in the study are publicly available. The RNA-seq data from this study have been deposited into NCBI SRA database with accession number PRJNA1006542 (https://www.ncbi.nlm.nih.gov/sra/?term=PRJNA1006542; last accessed on 17 February 2024).

## Supplementary Materials

The accompanying supplementary information is available for download at: https://www.mdpi.com/article/10.3390/plants13050596/s1, Figure S1: The *ossnrk1a* mutants displayed no obvious defects in grain size and weight. Figure S2: Phenotypes of ZH11 and *ossnrk1a*−*1* under starvation stress. Figure S3: The *ossnrk1a* mutants showed more susceptibility to *M. orzyae* infection. Figure S4: Correlation analysis of the transcriptome replicates and Venn plots of the DEGs in *ossnrk1a*−*1* and ZH11. Figure S5: Transcript levels of *OsSnRK1a*, *OsSnRK1β1* and *OsSnRK1β3* in leaf, young panicle and root tissues of ZH11 at heading stage; Table S1: List of primer pairs used in this study. Table S2: Gene ID, FPKM, and functions of DEGs in Figure 3 and Figure 4. Supplementary File S2 shows two biological replicates.

## Abbreviations

AOS2	Allene oxide synthase 2
BP	Biological processes
CC	Cellular components
Co-IP	Co-immunoprecipitation
DEGs	Differently expressed genes
GFP	Green fluorescence protein
GO	Gene Ontology
ha.	Hectare
hpi	Hours post inoculation
IH	Invasive hyphae
JA	Jasmonic acid
MF	Molecular functions
PAMP	Pathogen-associated molecular pattern
PTI	PAMP-triggered immunity
qRT-PCR	Real-time quantitative reverse transcription PCR
ROS	Reactive oxygen species
SA	Salicylic acid
SnRK1	Sucrose non-fermenting-1-related protein kinase-1
Y2H	Yeast two-hybrid
YFP	Yellow fluorescent protein
ZT	Zeitgeber time
