# Supplementary material for: Identification of Ossnrk1a−1 Regulated Genes Associated with Rice Immunity and Seed Set
Source: Plants (Basel). 2024 Feb 22;13(5):596. doi: 10.3390/plants13050596 (PMC10935424; doi:10.3390/plants13050596)
Supplement: Supplementary file 1 [file plants-13-00596-s001.zip › plants-2837071-supplementary file S1.pdf]

Supplementary file S1

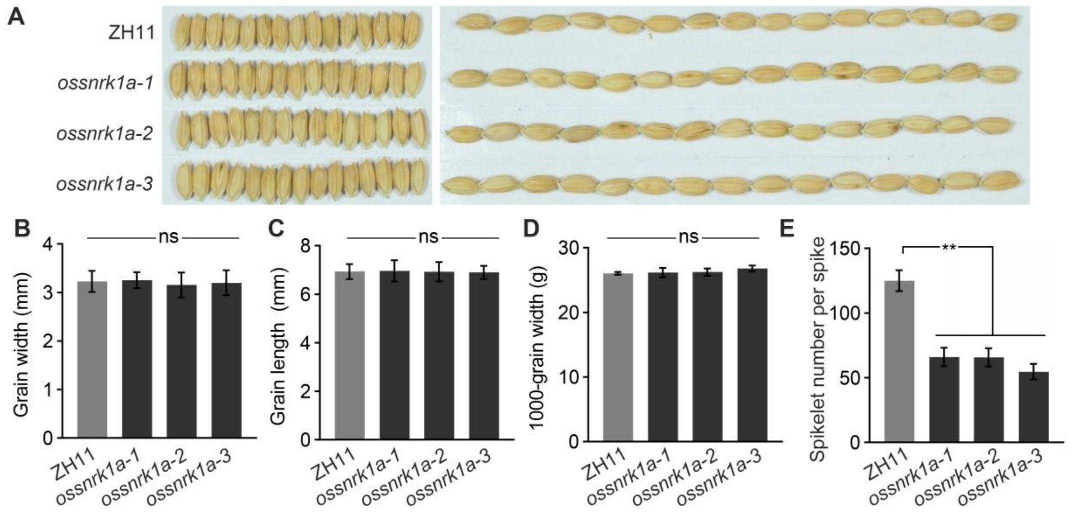

Figure S1. The *ossnrk1a* mutants displayed no obvious defects in grain size and weight, compared with the wild type. **(A)** Morphology of mature grains from ZH11 and the *ossnrk1a* mutants. **(B)** and **(C)** Measurement of grain width, and grain length of ZH11 and *ossnrk1a* mutant seeds, respectively. with bars representing mean values ± SD (n = 15). **(D)** 1000-grain weight of ZH11 and *ossnrk1a* mutants with bars representing means ± SD (n = 3). **(E)** The spikelet number per spike of ZH11 and *ossnrk1a* mutants with bars representing means ± SD. "ns" means no significance by Student's *t*-test.

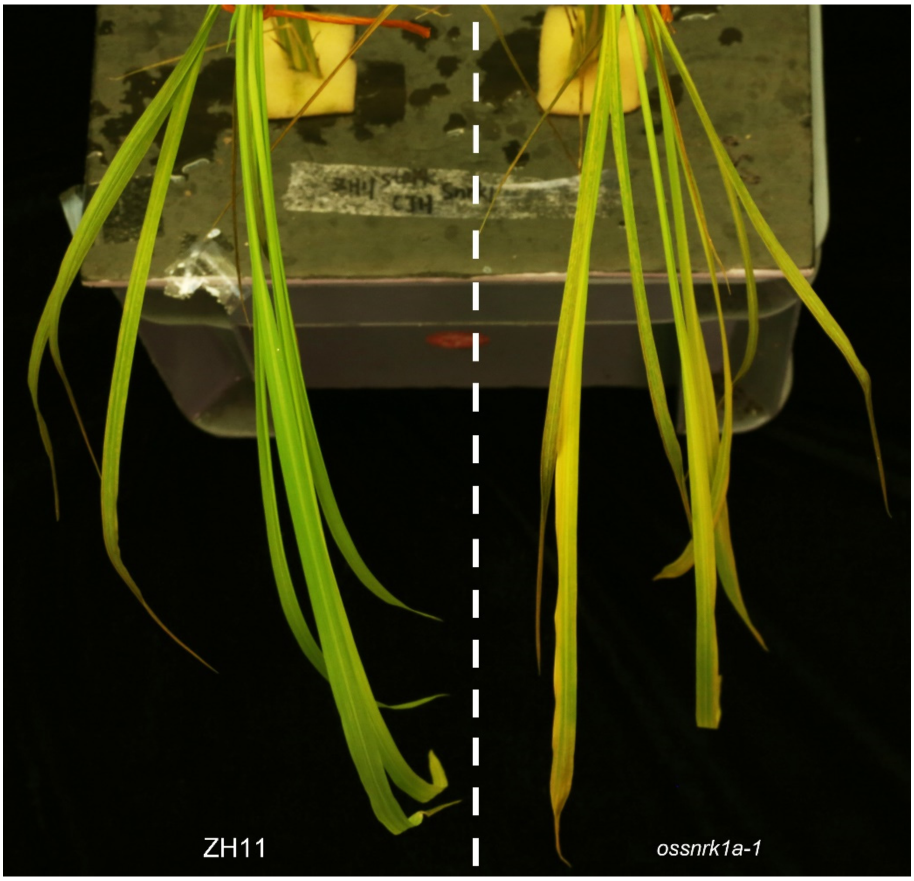

Figure S2. Phenotypes of ZH11 and *ossnrk1a-1* under starvation/continuously dark stresses. After 4-day continuously dark treatment, the water-culturing ZH11 (left) and *ossnrka-1* (right) plants were imaged.

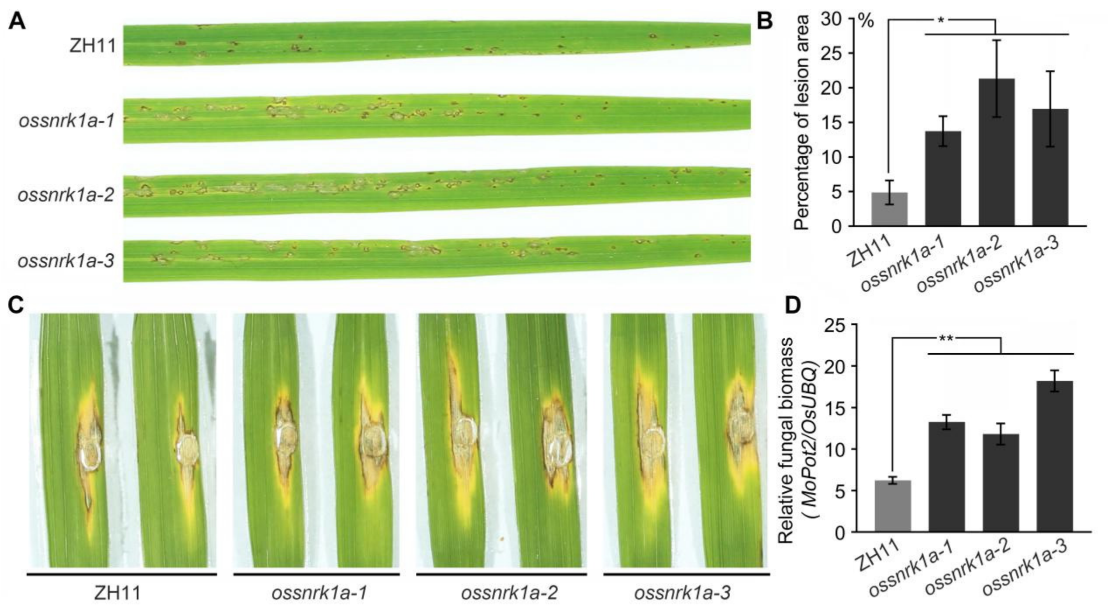

Figure S3. The *ossnrk1a* mutants showed more susceptibility to *M. orzyae* infection. **(A)** and **(B)** Spraying inoculation on 3-week-old ZH11 and *ossnrk1a* mutants seedlings with Guy11 conidia. The leaves of *ossnrk1a* mutants at 5 dpi displayed larger diseased lesion area, compared to those of ZH11. **(C)** Five-week-old plants of ZH11 and *ossnrk1a* mutants were subjected to punch inoculation with Guy11 conidia. The diseased leaves were photographed at 9 dpi. **(D)** Fungal biomass of the punch-inoculation leaves was measured by qPCR analysis (*MoPot2*/*OsUBQ*) with extracted genomic DNA as templates. Bars in **(B)** and **(D)** are means ± SD from three biological replicates. Significant differences were determined by Student's *t*-test (*, $p < 0.05$ and **, $p < 0.01$).

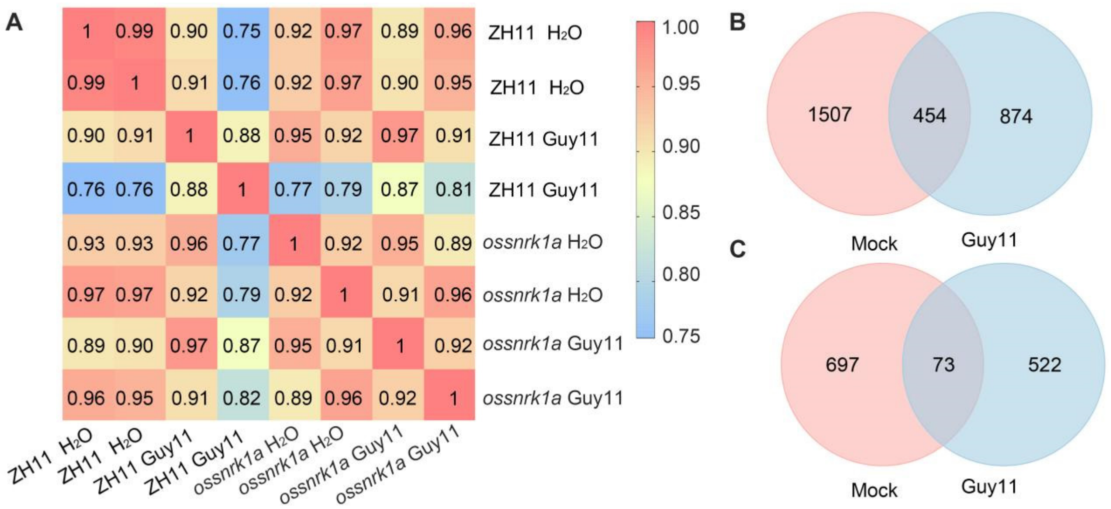

Figure S4. Correlation analysis of the transcriptome replicates and venn plots of the DEGs in *ossnrk1a-1* and ZH11. **(A)** Correlation analysis using Pearson correlation coefficient indicating high correlation coefficients of the biological replicates for each sample in RNA-seq. **(B)** and **(C)** are the venn diagrams indicating the upregulated (B) and downregulated (C) DEGs numbers from *ossnrk1a-1* vs. ZH11 with non-pathogen (pink pie) and Guy11 infection treatments (blue pie), respectively.

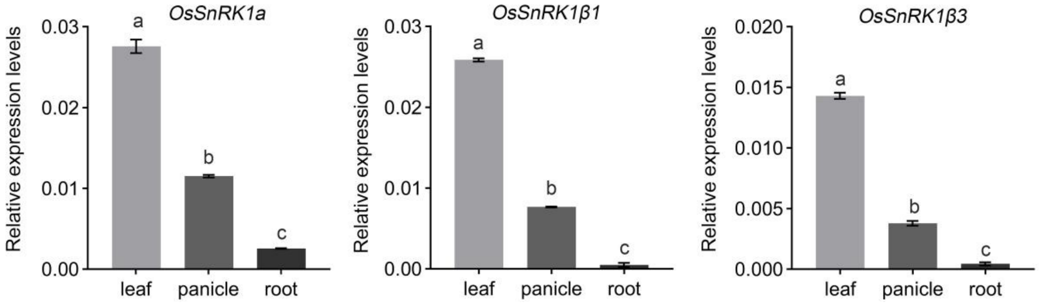

Figure S5. Transcript levels of *OsSnRK1a*, *OsSnRK1β1* and *OsSnRK1β3* in leaf, young panicle and root tissues of ZH11 at heading stage. Data are means ± SD (n = 3). Statistically significant differences were indicated by Mann-Whitney test (Different letters mean significant difference among treatments ($p < 0.05$)).

Table S1. List of primer pairs used in this study.

Plasmid construction		
Gene	Forward Sequence (5′-3′)	Reverse sequence (5′-3′)
OsSnRK1a-GFP	ACTGGGATCCATGGAGGGAGCTGGCAGAGA	ACTGGAATTCAAGGACTCTCAGCTGAGTTA
OsSnRK1β1-GFP	CGCTCTAGAACTAGTGGATCCATGGGGAACGCGAGCGGGC	GATAAGCTTGATATCGAATTCTCGTTCAATGGGCTTATACAG
OsSnRK1β3-GFP	ACTGGGATCCATGGGGAACGCGAGCGGCAA	ACTGGTCGACACCACCATTCTTTGTTCGCTTC
OsSnRK1a-HA	ACTGGGATCCATGGAGGGAGCTGGCAGAGA	ACTGGAATTCAAGGACTCTCAGCTGAGTTA
OsSnRK1a-nLUC	GGGGACAAGTTTGTACAAAAAAGCAGGCTTCATGGAGGGAGCTGGCAGAGA	GGGGACCACTTTGTACAAGAAAGCTGGGTCAAGGACTCTCAGCTGAGTTA
OsSnRK1β1-cLUC	GGCGGTACCCGGGATCCAATGGGGAACGCGAGCGGGC	GAAAGCTCTGCAGGTCGACCTAtCGTTCAATGGGCTTATACAG
OsSnRK1β3-cLUC	GGGGACAAGTTTGTACAAAAAAGCAGGCTTCATGGGGAACGCGAGCGGCAA	GGGGACCACTTTGTACAAGAAAGCTGGGTCACCACCATTCTTTGTTCGCTTC
pGADT7-OsSnRK1β1	GTACCAGATTACGCTCATATGATGGGGAACGCGAGCGGGC	ATGCCCACCCGGGTGGAATTCCTACTATCGTTCAATGGGCTT

		ATACAG
pGADT7-OsSnRK1β3	ACTGCATATGATGGGGAACGCGAGCGGCAA	ACTGGGATCCTTAACCACCATTCTTTGTTC
qRT-PCR		
Gene	Forward Sequence (5′-3′)	Reverse sequence (5′-3′)
OsAOS2	CGTCCAAAGTTTCGGGAGTT	CTCCATGGCGCCTAGCTAA
OsJAMyb	GAGGACCAGAGTGCAAAAGC	CATGGCATCCTTGAACCTCT
osWRKY45	GGACGCAGCAATCGTCCGGG	CGGAAGTAGGCCTTTGGGT
OsNADHGOGAT2	CCTGTCGAAGGATGATGAAGGTGAAACC	TGCATGGCCCTACTATCTTCGCATCA
OsPR1b	ACGGGCGTACGTACTGGCTA	CTCGGTATGGACCGTGAAG
OsSnRK1β1	TGTGGACGCTTGGAGGAAA	CCGATGGAAGTATCAACAGGAG
OsSnRK1β3	GGACATCAAGGAGGGTGTTAGA	CATCAGCCACATGAGGTAGTTC
OsSnRK1a	CGAATCACTTCACAAGAGACTG	CTGGAGTTACTTGAGCGAGAG
OsUBQ	TTCTGGTCCTTCCACTTTCAG	ACGATTGATTTAACCAGTCCATGA
MoPot2	ACGACCCGTCTTTACTTATTTGG	AAGTAGCGTTGGTTTTGTTGGAT

Table S2. Gene ID, FPKM, and functions of DEGs in Fig 3 and Fig 4.

GO name	Gene ID	FPKM								Functions
		ZH11_H_2O （Mock）		ZH11_Guy11 (48 hpi）		ossnrk1a-1_H_2O （Mock）		ossnrk1a-1_Guy11 (48 hpi）		
response to biotic stimulus	LOC_Os06g34400	7.97	7.38	23.86	14.45	8.59	5.75	6.57	6.95	zinc finger, C3HC4 type domain containing protein
	LOC_Os07g08300	16.81	13.14	29.94	15.46	14.25	9.91	15.58	12.47	major facilitator superfamily antiporter, putative, expressed
	LOC_Os01g41750	0.88	0.79	2.50	2.84	1.31	0.94	1.42	0.97	expressed protein
	LOC_Os05g29710	0.66	1.11	11.15	1.87	0.66	0.62	0.97	0.77	RING-H2 finger protein, putative, expressed
	LOC_Os03g03700	0.56	0.21	4.12	0.14	0.55	2.82	2.15	1.61	MLO domain containing protein, putative, expressed
	LOC_Os05g28770	0.49	0.57	2.28	1.64	0.60	0.36	0.57	0.64	GCRP9 - Glycine and cysteine rich family protein precursor, expressed
	LOC_Os01g713	2.64	7.92	39.98	27.70	25.39	5.29	20.13	13.54	glycosyl hydrolases family 17, putative,

	50									expressed
	LOC_Os10g39700	0.65	1.39	1.72	3.28	2.57	0.91	1.26	0.84	CHIT15 - Chitinase family protein precursor, putative, expressed
	LOC_Os05g51610	3.49	3.68	24.34	8.59	3.16	2.10	5.56	5.64	sodium/calcium exchanger protein, putative, expressed
	LOC_Os10g13700	1.53	1.13	3.84	2.45	1.09	1.04	1.50	1.08	phosphoenolpyruvate carboxykinase, putative, expressed
	LOC_Os03g55670	53.14	81.03	358.81	112.07	70.03	67.68	96.48	60.93	expressed protein
	LOC_Os07g13100	1.41	2.73	9.18	2.37	1.72	1.47	1.19	1.48	DNA binding protein, putative, expressed
	LOC_Os12g44070	0.04	0.00	2.35	1.15	0.38	0.32	0.43	0.19	nodulin, putative, expressed
	LOC_Os05g45980	0.83	0.67	2.07	1.08	0.56	0.37	0.64	0.61	expressed protein
	LOC_Os02g10120	7.09	7.68	4.98	5.20	3.69	5.64	1.19	0.96	lipoxygenase gene
	LOC_Os03g23050	0.17	0.00	0.29	0.20	0.05	0.07	0.16	0.04	expressed protein
	LOC_Os01g713	4.27	20.65	96.84	161.8	50.50	22.74	56.68	22.38	glycosyl hydrolases family 17, putative,

	40				9					expressed
	LOC_Os01g28450	0.11	0.43	2.25	2.01	0.92	0.00	1.10	1.01	pathogenesis-related protein 1b，OsPR1b
Energy metabolism	LOC_Os10g21212	0.88	1.03	1.09	0.68	0.81	0.36	0.93	0.79	photosystem II 44 kDa reaction center protein, putative, expressed
	LOC_Os10g21310	2.65	1.53	2.44	2.29	2.31	1.06	1.14	1.78	photosystem II P680 chlorophyll A apoprotein, putative, expressed
	LOC_Os04g16770	16.13	11.80	17.93	10.96	12.90	4.17	8.10	10.86	photosynthetic reaction center protein, putative, expressed
	LOC_Os01g64120	49.73	35.61	354.79	124.09	29.86	10.69	30.21	38.88	Ferredoxin，OsFd5
	LOC_Os08g15322	0.72	0.38	0.73	0.64	0.79	0.32	0.15	0.34	cytochrome b559 subunit alpha, putative, expressed
	LOC_Os10g13700	1.53	1.13	3.84	2.45	1.09	1.04	1.50	1.08	phosphoenolpyruvate carboxykinase, putative, expressed
	LOC_Os08g10020	67.21	25.69	46.05	84.54	61.70	95.72	67.62	27.98	photosystem II 10 kDa polypeptide, chloroplast precursor,
	LOC_Os05g48200	31.19	37.52	103.53	42.72	16.17	7.87	21.02	13.19	NADH-glutamate synthase 2 gene, *OsNADH-GOGAT2*
	LOC_Os01g580	13.55	24.04	25.47	8.76	8.06	4.50	22.04	9.12	NADPH-dependent oxidoreductase, putative,

Energy metabolism	22									expressed
	LOC_Os10g21324	2.06	1.47	2.35	1.63	0.70	1.26	1.61	0.91	cytochrome b6, putative, expressed
	LOC_Os05g48200	31.19	37.52	103.53	42.72	16.17	7.87	21.02	13.19	NADH-glutamate synthase 2 gene, *OsNADH-GOGAT2*
	LOC_Os02g53130	23.05	2.88	6.07	9.26	2.08	1.52	2.35	4.44	nitrate reductase gene
	LOC_Os08g14860	6.32	7.19	22.08	8.03	5.39	7.55	13.59	4.611	Hydroxyacid oxidase 1
	LOC_Os11g13890	513.37	318.09	49.27	387.65	306.84	330.30	215.02	155.31	light-harvesting complex II protein
	LOC_Os10g38276	2.50	2.19	0.97	0.42	1.09	0.47	1.10	0.60	chloroplast ATP synthase a chain precursor, putative, expressed
	LOC_Os01g57945	8.00	5.35	6.70	4.14	2.40	2.41	4.57	2.93	cytochrome b6-f complex subunit 4, putative, expressed
	LOC_Os01g59920	15.49	10.64	4.17	8.25	6.18	2.91	2.06	3.85	cysteine synthase, chloroplast precursor, putative, expressed
	LOC_Os01g11054	12.20	7.67	13.90	12.02	8.98	4.31	11.10	11.15	phosphoenolpyruvate carboxylase
	LOC_Os10g212	2.97	1.52	1.55	1.61	1.13	0.44	1.17	1.57	ATP synthase subunit beta, putative, expressed

	66									
	LOC_Os04g16848	4.53	3.71	4.16	3.48	3.80	1.70	2.26	3.12	photosystem II reaction center protein H, putative
	LOC_Os08g36480	215.28	122.94	212.15	134.45	75.89	75.76	94.87	83.29	nitrate reductase
	LOC_Os12g44150	64.37	53.00	28.16	37.89	28.16	37.89	36.22	31.68	plasma membrane H+-ATPase, *OsA3*
	LOC_Os01g25484	6.45	4.09	13.55	6.03	4.50	0.66	1.72	2.31	leaf ferredoxin-nitrite reductase; promoter of shoot regeneration 1, *OsNiR1*
