# Supplementary material for: Identification of Ossnrk1a−1 Regulated Genes Associated with Rice Immunity and Seed Set
Source: Plants (Basel). 2024 Feb 22;13(5):596. doi: 10.3390/plants13050596 (PMC10935424; doi:10.3390/plants13050596)
Supplement: Supplementary file 1 [file plants-13-00596-s001.zip › plants-2837071-supplementary file S2.pdf]

Supplementary files S2: Results of the mentioned biological replicates in this study.

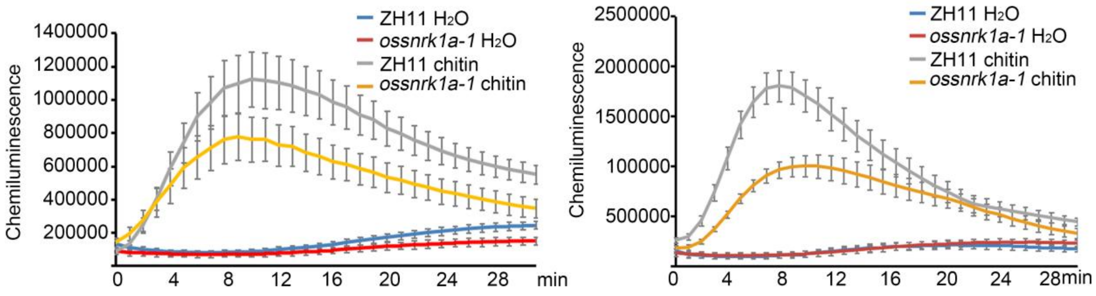

These results are the other two biological replicates of Figure 2C.

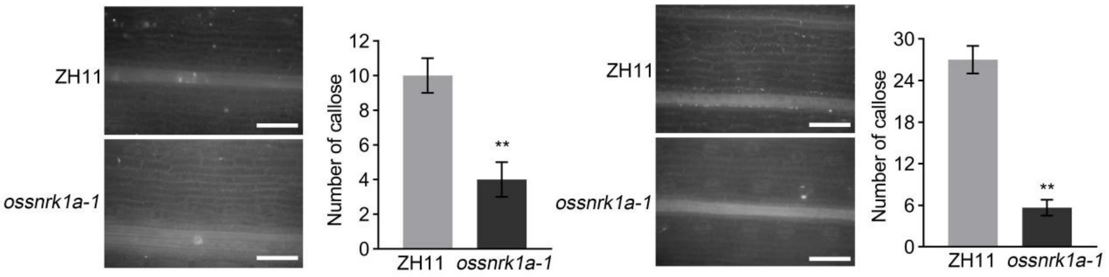

These results are the other two biological replicates of Figure 2D.

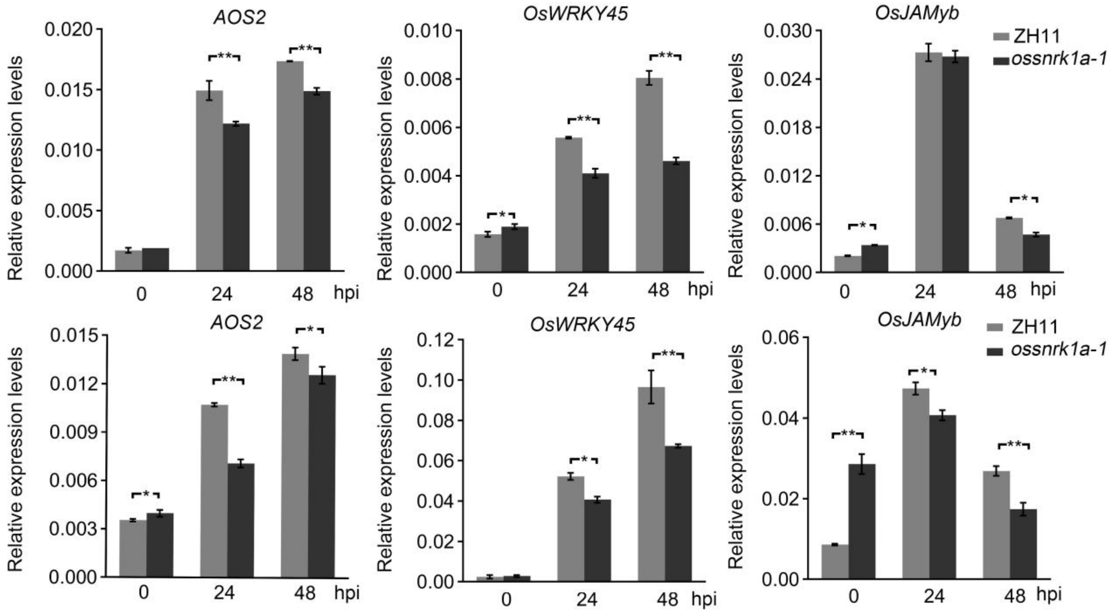

These results are the other two biological replicates of Figure 2F.

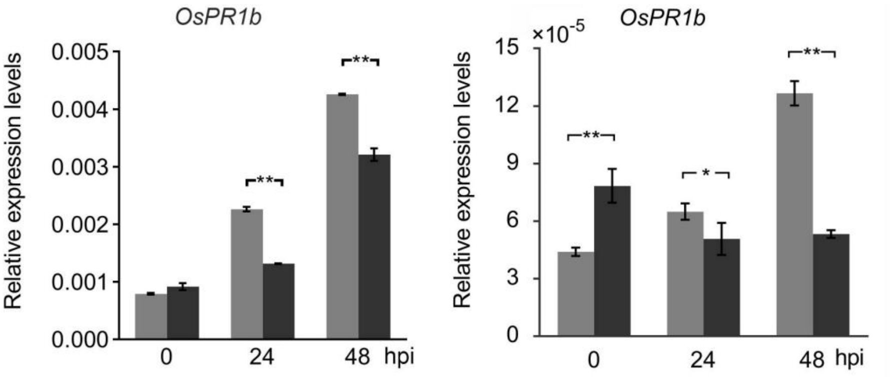

These results are the other two biological replicates of Figure 3C.

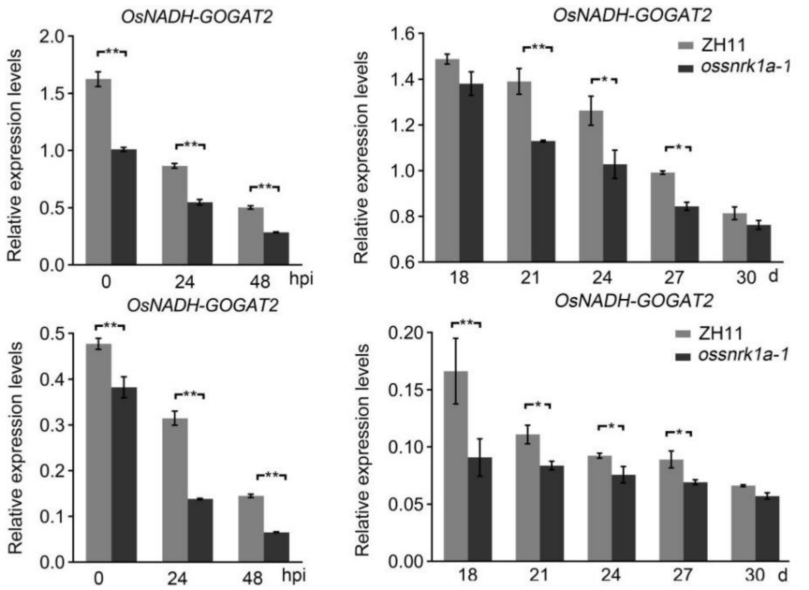

These results are the other two biological replicates of Figure 4E and 4F.

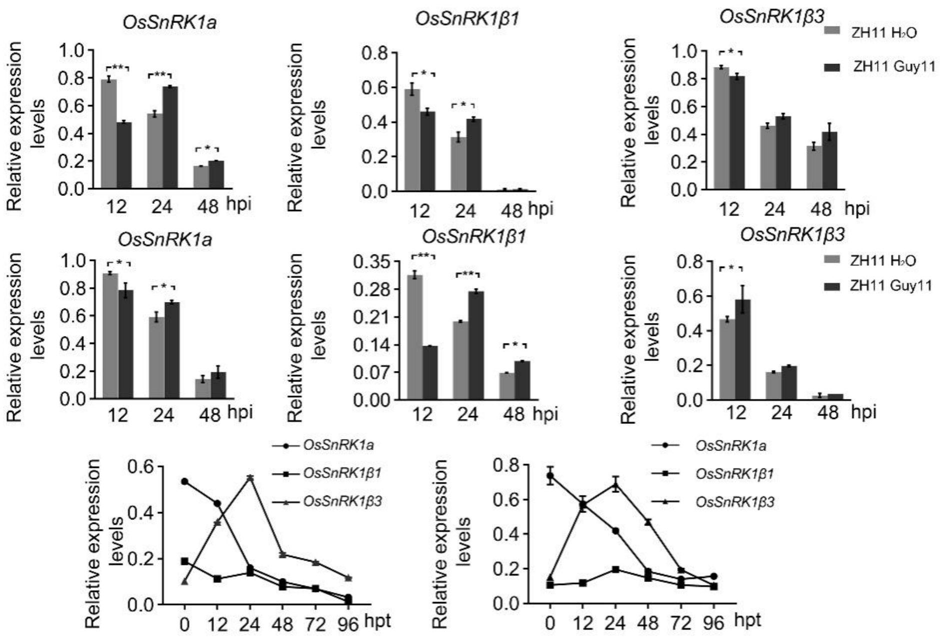

These results are the other two biological replicates of Figure 6B and 6C.

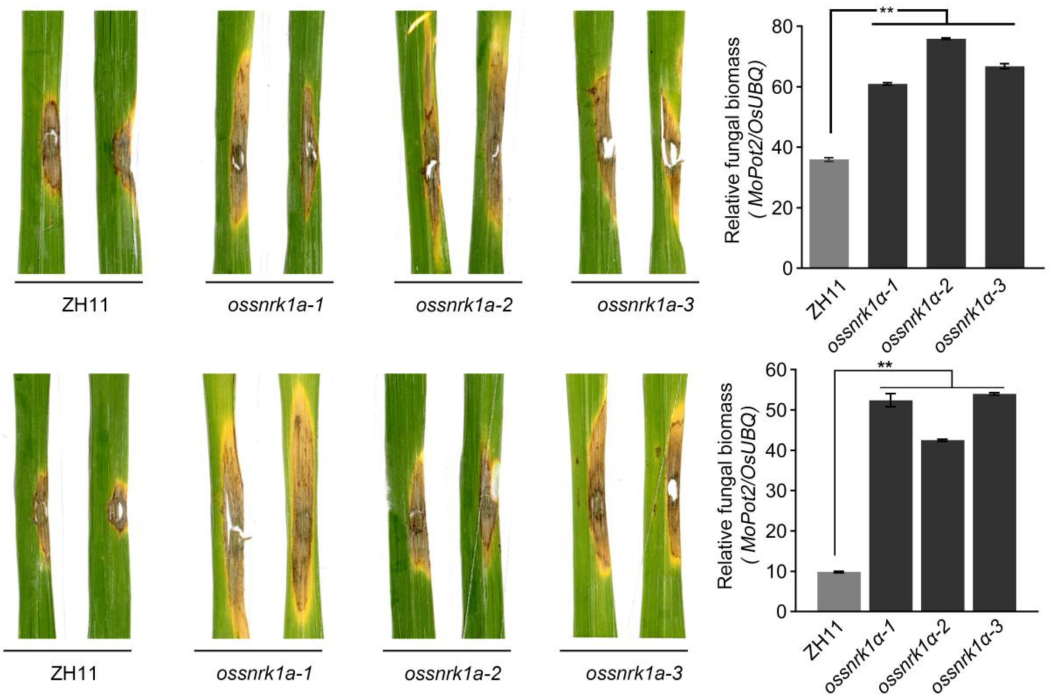

These results are the other two biological replicates of Figure S3C and S3D.

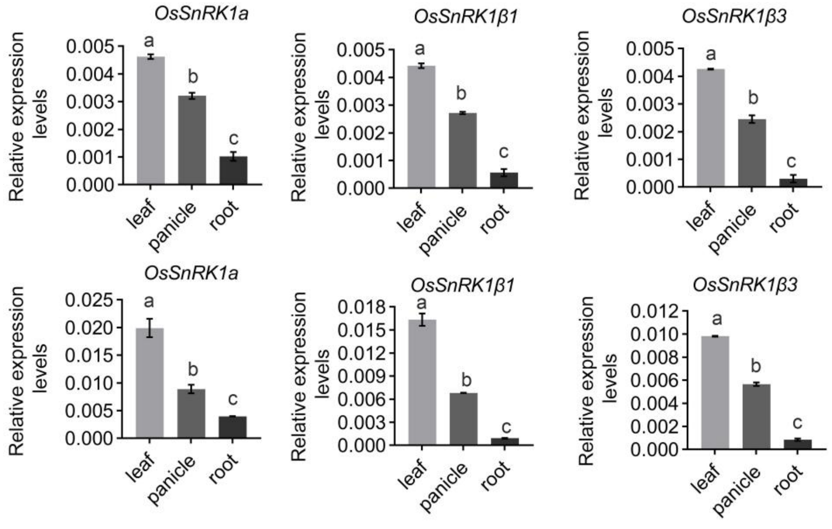

These results are the other two biological replicates of Figure S5.
